# Accuracy of the Freestyle Libre 3 Continuous Glucose Monitoring System in Hypo‐ and Euglycemic Cats

**DOI:** 10.1111/jvim.70048

**Published:** 2025-03-19

**Authors:** Antonio M. Tardo, Chiquitha Crews, Jocelyn Mott, Lauren T. Porter, Christopher Adin, Chen Gilor

**Affiliations:** ^1^ Department of Veterinary Medical Sciences University of Bologna Ozzano dell'Emilia Italy; ^2^ Department of Small Animal Clinical Sciences University of Florida, College of Veterinary Medicine Gainesville Florida USA

**Keywords:** diabetes mellitus, flash glucose monitoring system, hypoglycemia, insulin, interstitial glucose

## Abstract

**Background:**

The FreeStyle Libre 3 (FSL3) has several improvements compared to previous FreeStyle Libre systems, but its accuracy has not yet been determined in cats. In diabetic people, FSL3 offers increased accuracy, and its smaller size could be advantageous for use in veterinary patients.

**Objectives:**

Assess the accuracy of FSL3 in cats with experimentally induced hypoglycemia.

**Animals:**

Seven healthy, purpose‐bred cats.

**Methods:**

Hyperinsulinemic‐hypoglycemic clamps were performed. Interstitial glucose concentration (IG), measured by FSL3, was compared to blood glucose concentration (BG) measured by AlphaTrak2. Data were analyzed for all paired measurements (*n* = 474) and during stable BG (≤ 1 mg/dL/min change over 10 min). Pearson's r test, Bland–Altman test, and Parkes Error Grid analysis (EGA) respectively used to determine correlation, bias, and clinical accuracy.

**Results:**

Blood glucose concentration and IG correlated strongly (*r* = 0.86, *p* < 0.0001) in stable glycemia and moderately at all rates of change (*r* = 0.73, *p* < 0.0001). Analytical accuracy was not achieved, whereas clinical accuracy was demonstrated with 99%–100% of the results in zones A + B of the Parkes EGA. Interstitial glucose concentration underestimated BG in euglycemia and mild hypoglycemia (mean −11.7 ± 11.2, −5.5 ± 9.1, −1.5 ± 6.0 mg/dL in the ranges 91–120, 66–90, and 56–65 mg/dL, respectively), but overestimated BG in marked hypoglycemia (mean 6.3 ± 5.7, 15.7 ± 5.6 mg/dL in the ranges 46–55 and < 45 mg/dL, respectively).

**Conclusions:**

The FSL3 underestimates BG across most of the hypo‐euglycemic range but overestimates BG in marked hypoglycemia (< 55 mg/dL). Recognizing the proportional, glycemic‐dependent bias of FSL3 improves the safety of its clinical application in cats.

**Abbreviations**
BCS: body condition score
BG: blood glucose concentration
CGMS: continuous glucose monitoring system
DM: diabetes mellitus
EGA: error grid analysis
FSL1: freestyle libre 1
FSL2: freestyle libre 2
FSL3: freestyle libre 3
IG: interstitial glucose concentration
VAP: vascular access port
vPBGM: veterinary portable blood glucose meter

## 1 Introduction

Continuous glucose monitoring systems (CGMS) have revolutionized the management of diabetes mellitus (DM) in both human and veterinary medicine [1, 2]. These devices measure interstitial glucose concentrations (IG) on a minute‐by‐minute basis over days or weeks, decreasing blood sampling‐associated patient discomfort and markedly increasing information on IG fluctuations and trends [2, 3, 4]. Their use improves detection of hypoglycemia in both human [5, 6, 7] and veterinary patients [3], offering a way to address primary stressors reported by owners [8, 9].

The FreeStyle Libre (Abbott Laboratories Ltd., Chicago, Illinois) is the most commonly studied CGMS in veterinary patients. The accuracy of the first generation of the FreeStyle Libre (FSL1) has been evaluated previously in both healthy and diabetic cats [4, 10, 11, 12]. Interstitial glucose concentrations measured by the FSL1 correlate well with blood glucose concentrations (BG) in the eu‐hyperglycemic range, but interpretation in the hypoglycemic range is limited by very small sample sizes [4, 10, 11, 12]. The second generation of FreeStyle Libre (FSL2) was updated with a new glucose algorithm that provided improved accuracy across the measurement range in people, specifically at the low end of the dynamic range [13]. In purpose‐bred cats, we demonstrated that FSL2 underestimates BG throughout most of the hypo‐euglycemic range and generally overestimates BG in marked hypoglycemia (< 50 mg/dL) [14].

In 2020, a third generation of the device, FreeStyle Libre 3 (FSL3), was licensed for use in diabetic people. The FSL3 uses the same sensing technology as the FSL2 to measure IG. Like the FSL2, the FSL3 provides continuous IG readings every minute, as well as offering glucose trends and alerts [15]. However, the FSL3 has a one‐piece sensor applicator, and the sensor is approximately 70% smaller than the FSL1 or FSL2 [15]. Moreover, the FSL3 automatically sends results to a smartphone without requiring users to scan the sensor to obtain a glucose result. In a recent study, the FSL3 demonstrated accurate performance across the dynamic glycemic range in diabetic people [15]. In the low BG range (< 70 mg/dL), this device showed good accuracy with 95% of IG within ±20 mg/dL of the BG reference method, but the evaluation at glucose concentrations < 54 mg/dL was limited by the small numbers of IG‐BG pairs [15]. The performance and smaller size of the FSL3 could be advantageous in veterinary patients, and clinical trials are warranted. Most FSL validation studies in both healthy and diabetic cats have focused predominantly on the eu‐hyperglycemic range [4, 10, 11, 12], often overlooking the hypoglycemic range, which is critical for monitoring potentially life‐threatening conditions. Our objective was to determine the analytical and clinical accuracy of the FSL3 in cats with experimentally induced hypoglycemia.

## 2 Materials and Methods

### 2.1 Animals

Seven neutered, purpose‐bred, domestic shorthair cats (five female, two male) were included, with median (range) ages of seven (6–7) years. Median body weight was 4.9 kg (3.9–6.2 kg) and median body condition score was seven (6–8) on a nine‐point scale. Cats were group‐housed in facilities accredited by the Association for Assessment and Accreditation of Laboratory Animal Care International. All cats were socialized and acclimatized to catheter bandages and routine handling and restraint for at least 3 months before the start of the study. Extensive environmental enrichment was provided, including 1–3 h of daily interaction with humans and 24‐h access to various toys and climbing apparatus. Cats were fed commercial dry cat food (Envigo 2060 Teklad Global Cat Diet) ad libitum in a sufficient amount to maintain body weight. Cats were deemed healthy based on routine weekly physical examinations, annual blood tests (CBC and serum biochemistry panels), and the absence of clinical signs of disease. Experiments were performed at ambient temperatures between 20°C and 24°C in the cats' routine environment. All animal use was approved by the University of Florida Institutional Animal Care and Use Committee (protocol number 202011101).

### 2.2 Vascular Access Port, Peripheral Catheter, and FreeStyle Libre 3 Placement

A vascular access port (VAP, CompanionPort, CP 202 K, Norfolk Vet Products, Skokie, Illinois) was surgically placed under general anesthesia into the jugular vein of each cat at least 3 months before beginning the experiment. Vascular access port patency was maintained using weekly heparinized saline (10 U/mL) irrigation followed by a 0.5 mL (100 U/mL) heparin lock injection, which was aspirated and discarded before sample collection. The night before each experiment, a peripheral IV catheter (3/400 22‐24ga, Terumo Survet Surflo ETFE, Ontario, Canada) was placed in a cephalic vein and removed at the end of the procedure. This cephalic catheter was used exclusively for IV infusions. For placement of cephalic catheters, cats were sedated using dexmedetomidine (1–5 μg/kg, IV). Sedation was reversed using a dexmedetomidine‐equivalent volume of IM atipamezole (25–50 μg/kg) and the cats were monitored until fully recovered from sedation.

The FSL3 sensor (Abbott Laboratories Ltd., Chicago, Illinois) was placed, as previously described [11], at least 1 h before each procedure [11]. After application, each sensor was paired with a dedicated smartphone using the FreeStyle Libre 3 app (Abbott Diabetes Care Inc., Alameda, California).

### 2.3 Hyperinsulinemic‐Hypoglycemic Clamps

Controlled hypoglycemia (BG targets of 60 and 45 mg/dL, with 45 min at each target) was achieved using a modification of the hyperinsulinemic‐hypoglycemic clamp protocol previously described [14]. In brief, insulin was infused at a constant rate (0.30 U/kg/h in one cat and 0.15 U/kg/h in 6 cats) and dextrose was infused at a variable rate while measuring BG every 5 min and adjusting the dextrose infusion rate so that target glycemia levels were achieved. Additional blood sampling was performed at baseline and at each clamped BG as part of a separate study in which counter‐regulatory hormones were measured. Less than 24 mL of blood was drawn from each cat to account for these 13 samples (1.6 mL each) and all BG samples (0.05 mL each). This total volume accounts for ≤ 7% of total blood volume and therefore was deemed unlikely to affect the results of the study.

### 2.4 Accuracy of FSL3

The accuracy of the FSL3 was assessed by comparison to a veterinary portable blood glucose meter (vPBGM; AlphaTrak2, Blood Glucose Monitoring System, Zoetis, Parsippany, New Jersey) previously validated for use in cats with a BG range of 20–750 mg/dL and an intra‐assay coefficient of variation of 3.8% [16]. To compare IG measured with FSL3 to the BG obtained with vPBGM, paired samples were collected. Interstitial glucose concentration was recorded using a smartphone connected to the FSL3 at the same time each BG was measured using the vPBGM, and both were recorded as paired values. The same vPBGM was used for all cats. Glucose and insulin were infused exclusively via a peripheral cephalic IV catheter, whereas all blood sampling was performed using only the jugular VAP.

### 2.5 Data Analysis

Data were first analyzed from all time points in which concurrent measurement of BG and IG were available. Analysis was repeated on the data subset limited to stable BG to account for blood‐interstitium lag time [11, 12]. Stable glycemia was defined as a change in BG of ≤ 1 mg/dL/min over 10 min preceding sample acquisition. Average absolute change first was calculated between two consecutive BG measurements 5 min apart (using the formula: [BG(*t*_*i*_) BG(*t*_*i*−5_)]/5 in which *t*_*i*_ is time point *I* and *t*_*i*−5_ is the time point preceding it). Average change over 10 min then was calculated by averaging the rate of change in the previous two 5‐min periods. If a 5‐min interim data point was missing, the rate of change was averaged directly between two readings 10 min apart ([BG(*t*_*i*_) BG(*t*_*i*−10_)]/10). Such was the case in a single IG reading per cat, for a total of 7 data points throughout the entire data set. Hypoglycemia was defined as a BG measurement < 60 mg/dL [17].

Data were assessed for normal distribution using the Shapiro–Wilk test. The correlation between BG and IG was assessed using Pearson's r test and differences between glycemic groups compared using analysis of variance (ANOVA) with Dunnett correction for multiple comparisons (with the 91–120 mg/dL glycemic range as control). Data homogeneity of variance was verified using Brown‐Forsythe and Bartlett's tests. Proportional bias between BG and IG measurements was assessed using the Bland–Altman test. Accuracy was assessed according to ISO 15197:2013 guidelines, with acceptable analytical accuracy defined as 95% of IG results being within 15 mg/dL (when BG ≤ 100 mg/dL) or 15% (when BG > 100 mg/dL) of paired BG, and clinical accuracy as ≥ 99% of IG falling in zones A or B of the Parkes Error Grid analysis (EGA) as formulated most strictly for people with Type I DM [18]. Statistical significance was set at *p* < 0.05.

## 3 Results

**FIGURE 1:**
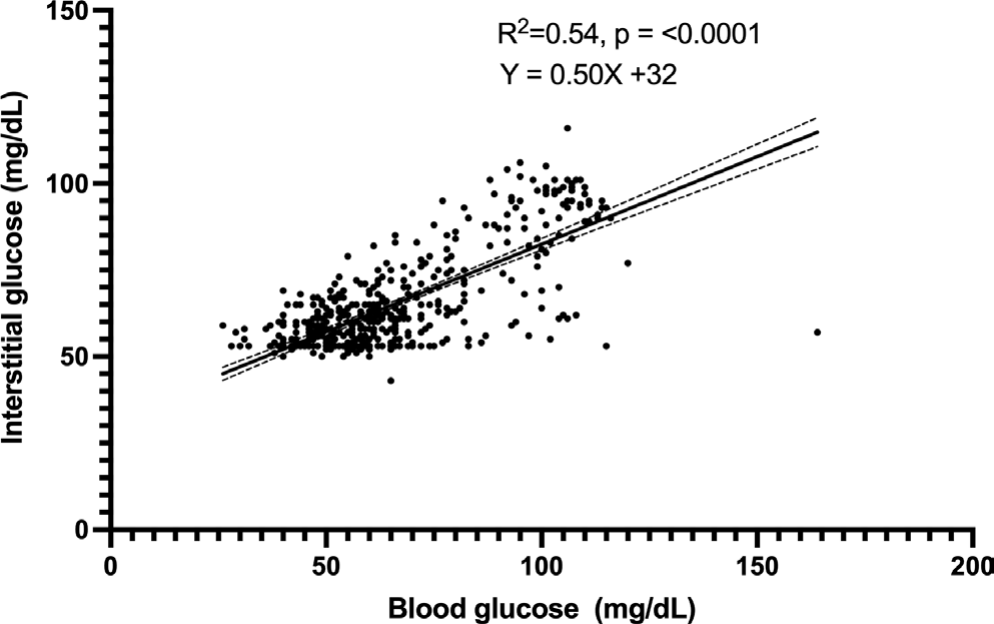
Correlation between blood glucose (BG) and interstitial glucose (IG) concentrations in healthy cats (*n* = 7) in hypo‐ and euglycemia at all rates of glycemic change. The solid line represents the best fit, with dashed lines representing the 95% CI of the best fit. Blood glucose is measured by AlphaTrak2. Interstitial glucose is measured by FreeStyle Libre 3.

**FIGURE 2:**
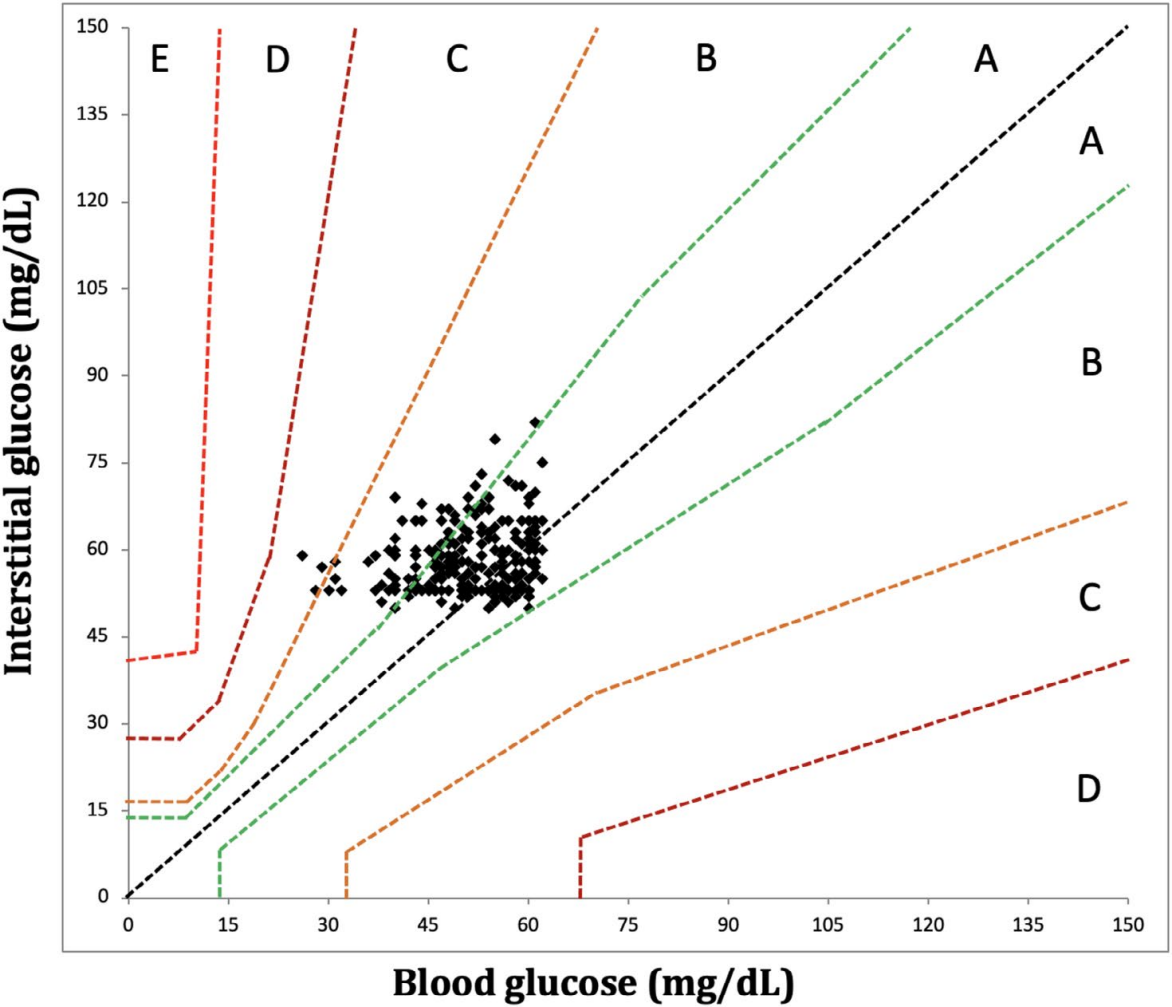
Parkes Error Grid analysis exhibiting excellent clinical accuracy of FSL3 at hypo‐ and euglycemia in healthy cats (*n* = 7) at all rates of glycemic change. Ninety‐nine percent of data points fall within zone A (indicating no change in clinical action) or zone B (indicating change in clinical action unlikely to affect outcome), with 90% (*n* = 427) in A and 9% (*n* = 46) in B. BG, blood glucose as measured by AlphaTrak2. IG, interstitial glucose as measured by FSL3, FreeStyle Libre 3.

**FIGURE 3:**
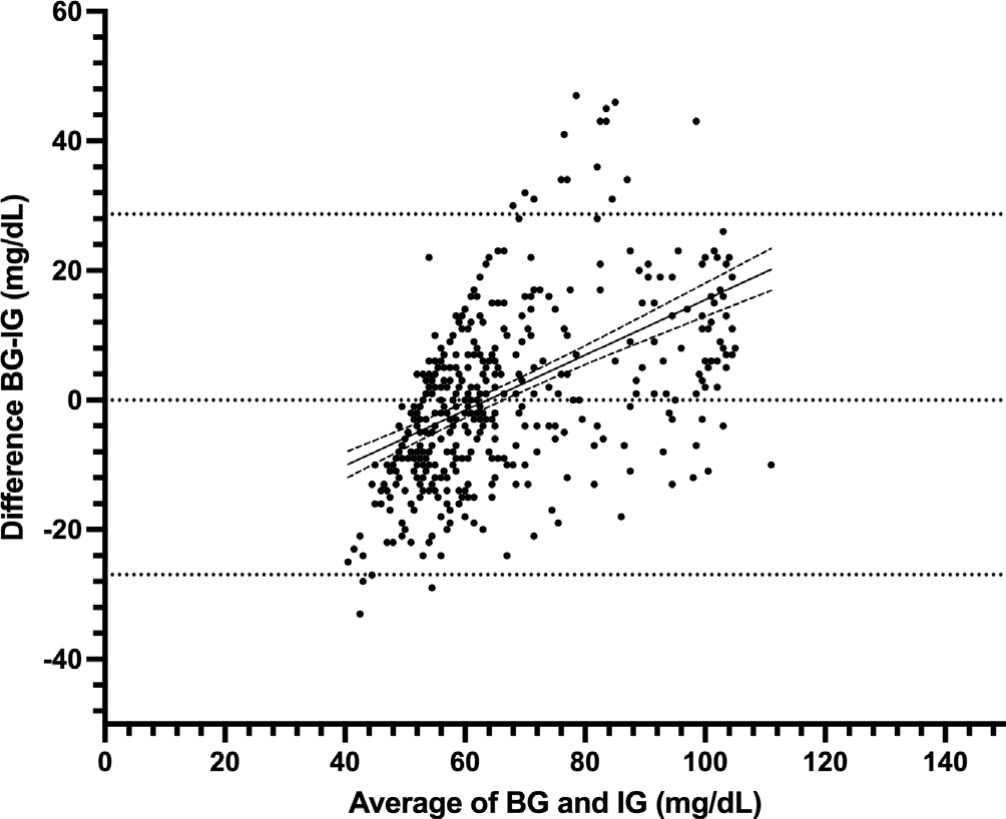
Bland Altman plot of agreement between blood glucose (BG) and interstitial glucose (IG) concentrations in hypo‐ and euglycemia in healthy cats (*n* = 7) at all rates of glycemic change. The middle, solid line represents the best fit, with dashed lines representing the 95% CI of the best fit. Blood glucose measured by AlphaTrak2. Interstitial glucose measured by FreeStyle Libre 3.

A total of 474 paired BG and IG data points were recorded from seven purpose‐bred cats during periods of hypoglycemia and euglycemia (range, 26–164 mg/dL). Of those paired values, 324/474 (68%) were in the low glucose range (BG < 70 mg/dL) and 223/474 (47%) in the hypoglycemic range (BG < 60 mg/dL). During periods of both stable and unstable glycemia (*n* = 474), BG and IG correlated moderately (*r* = 0.73, 95% confidence interval [CI], 0.69–0.77; *p* < 0.0001; Figure 1). In the Parkes EGA, 99% of IG results fell in zones A and B (427 in zone A [90%], 46 in zone B [9%]), and 1% (4) of values in zone C (Figure 2). In total, 375/474 (79%) of all IG results were within 15 mg/dL (when BG ≤ 100 mg/dL) or 15% (when BG > 100 mg/dL) of their paired BG results. Overall bias between BG and IG was 0.9 ± 14.2 (95% CI, −26.9 to 28.7) mg/dL (Figure 3).

**FIGURE 4:**
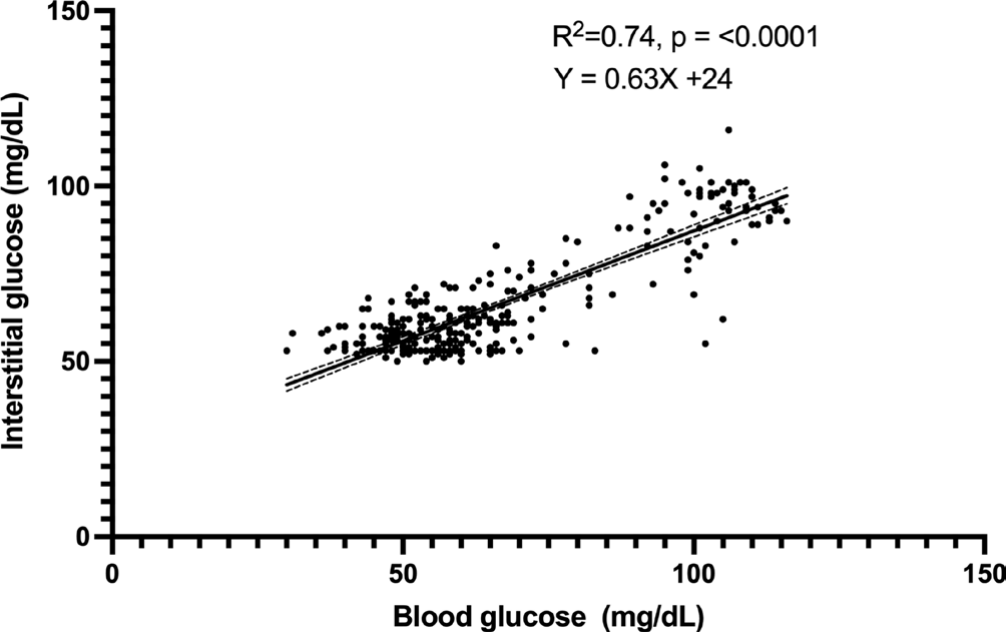
Correlation between blood glucose (BG) and interstitial glucose (IG) concentrations in healthy cats (*n* = 7) in hypo‐ and euglycemia during stable BG. The middle solid line represents the best fit, with dashed lines representing the 95% CI of the best fit. Blood glucose is measured by AlphaTrak2. Interstitial glucose is measured by FreeStyle Libre 3.

**FIGURE 5:**
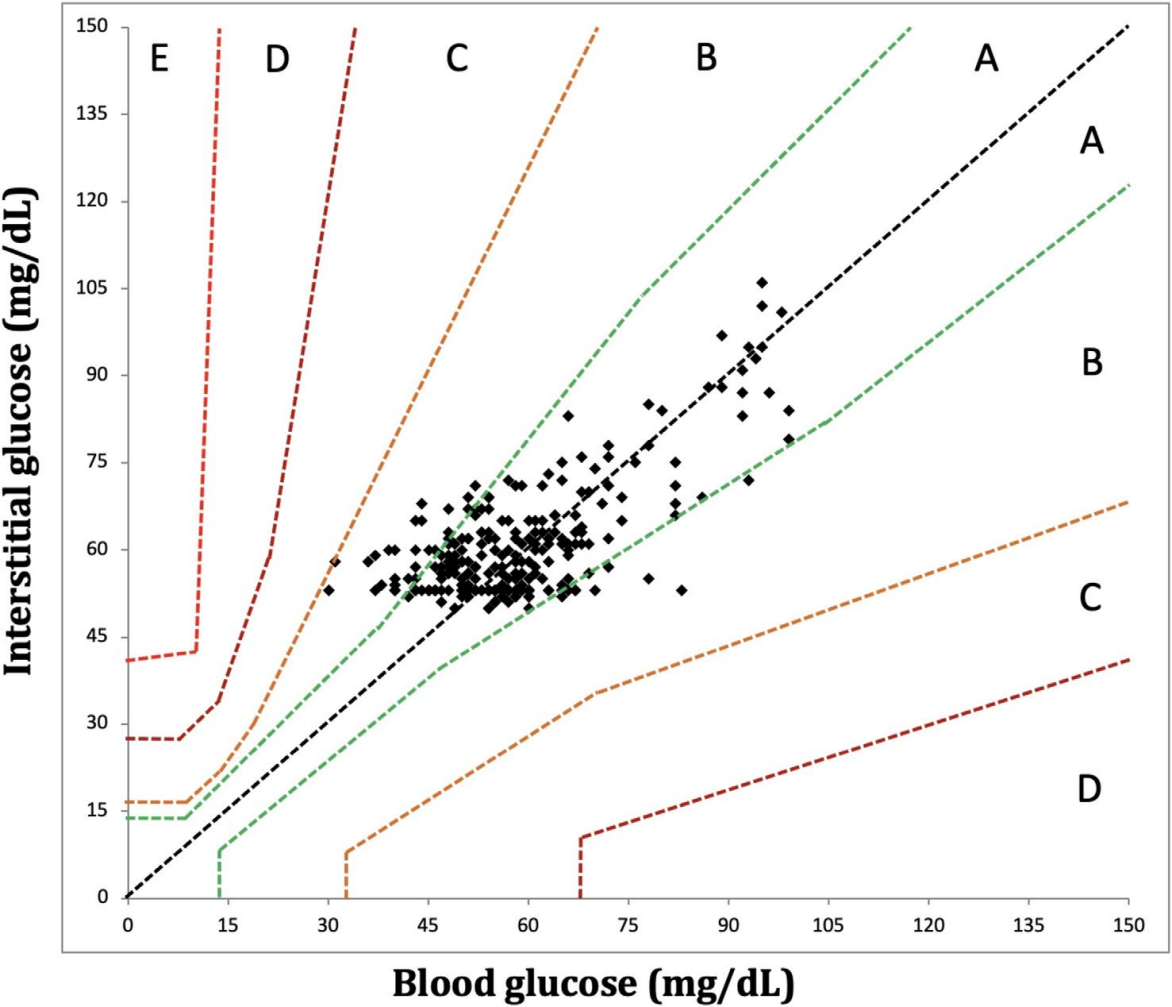
Parkes Error Grid analysis exhibiting excellent clinical accuracy of FSL3 at hypo‐ and euglycemia in healthy cats (*n* = 7) during stable blood glucose (BG) concentrations. All data points fall within zone A (indicating no change in clinical action) or zone B (indicating change in clinical action unlikely to affect outcome), with 90% (*n* = 272) data points in zone A and 10% (*n* = 29) in zone B. BG, measured by AlphaTrak2. IG, interstitial glucose measured by FreeStyle Libre 3.

**FIGURE 6:**
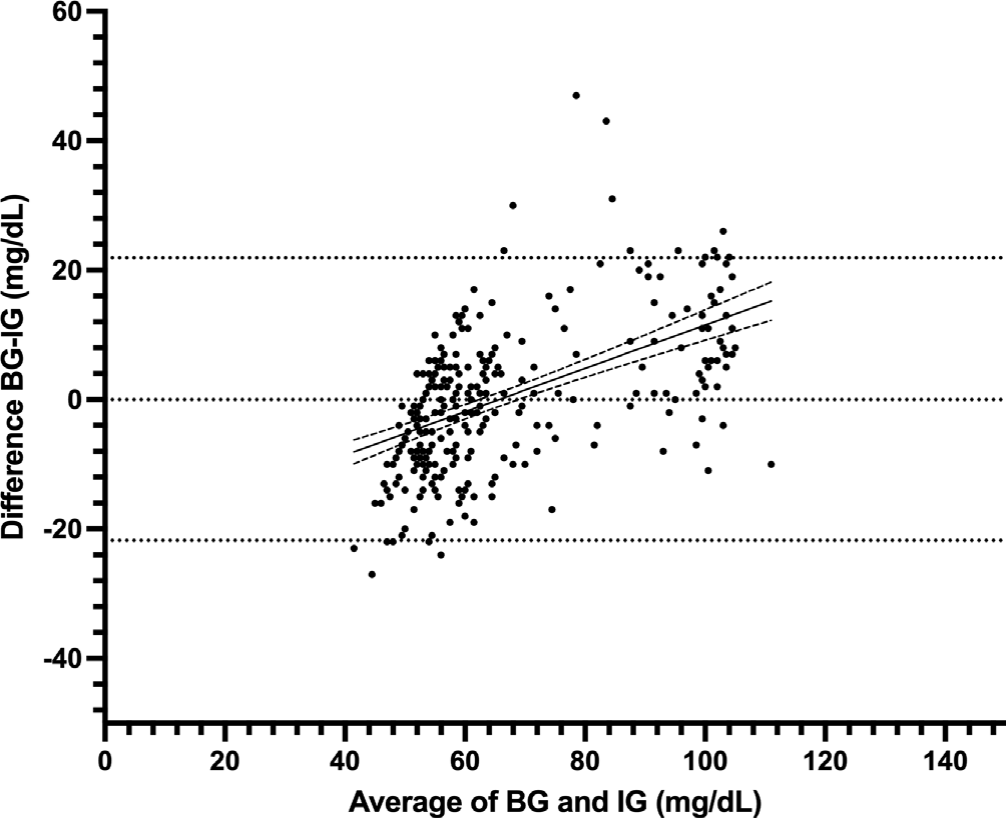
Bland‐Altman plot of agreement between blood glucose (BG) and interstitial glucose (IG) concentrations in hypo‐ and euglycemia in healthy cats (*n* = 7) during stable BG. The solid line represents the best fit, with dashed lines representing the 95% CI of the best fit. BG, blood glucose as measured by AlphaTrak2. IG, interstitial glucose as measured by FreeStyle Libre 3.

**FIGURE 7:**
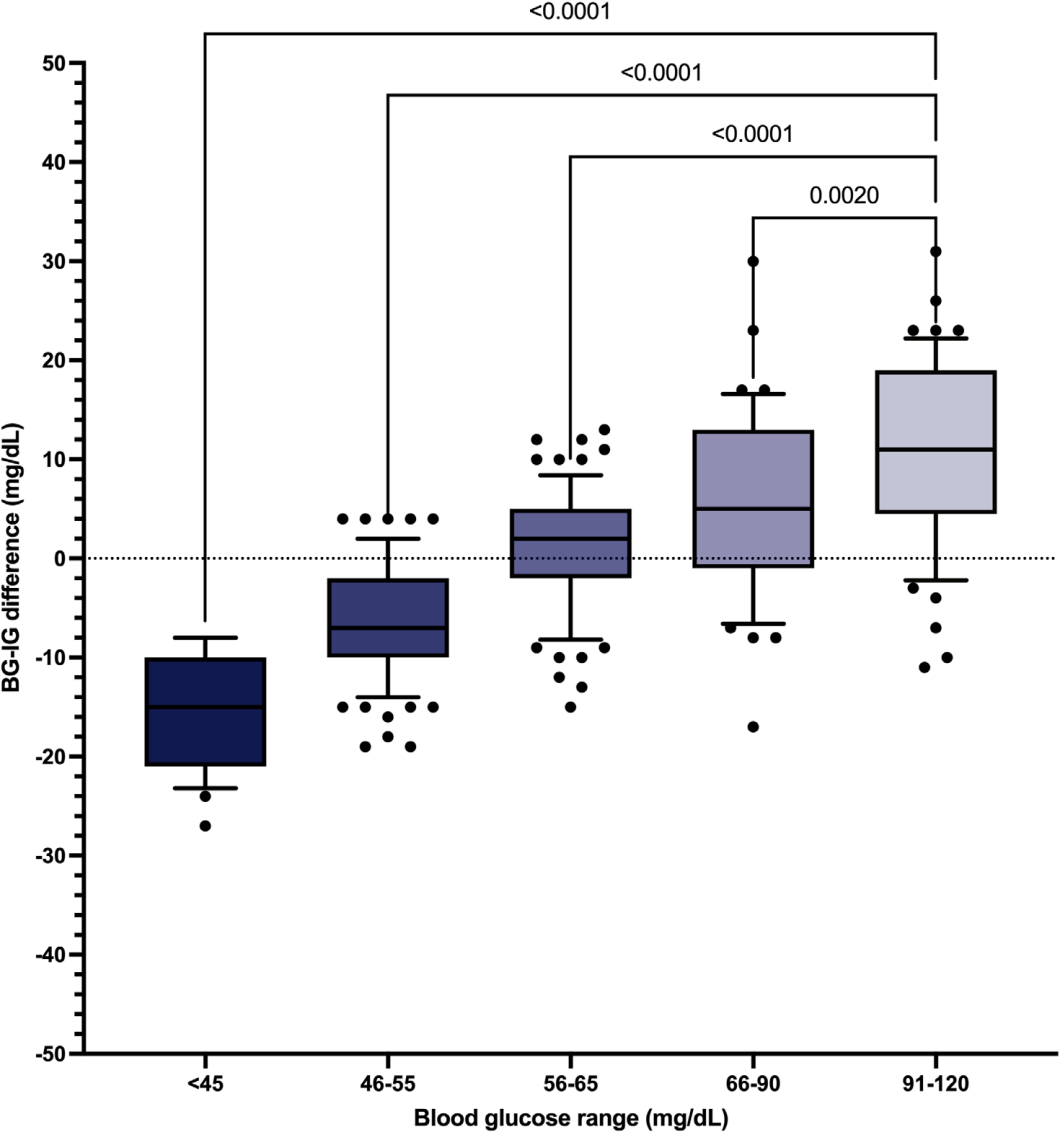
Difference between blood glucose (BG) and interstitial glucose (IG) concentrations, stratified based on BG range in healthy cats (*n* = 7) during stable BG. Central horizontal lines represent median values, boxes represent quartiles, and whiskers represent 10%–90% percentiles.

Of the entire data set of 474 pairs, 301 paired results occurred during periods of stable glycemia (≤ 1 mg/dL/min change in BG over 10 min), including 218/301 (72%) results in the low BG range (< 70 mg/dL) and 160/301 (53%) in the hypoglycemic range (BG < 60 mg/dL). In this subset, BG and IG correlated strongly (*r* = 0.86; 95% CI, 0.83–0.89; *p* < 0.0001; Figure 4) and 100% of IG results were in zones A and B of the Parkes EGA. Of these, 272 pairs were in zone A (90%) and 29 in zone B (10%; Figure 5). In total, 261/301 (87%) of all IG results were within 15 mg/dL (when BG ≤ 100 mg/dL) or 15% (when BG > 100 mg/dL) of their paired BG results. Overall bias between BG and IG was −0.07 ± 11.1 (95% CI, −21.9 to 21.8) mg/dL (Figure 6). Interstitial glucose concentration underestimated BG in euglycemia and mild hypoglycemia by a mean of 11.7 ± 11.2 mg/dL in the 91–120 mg/dL range (*n* = 59), 5.5 ± 9.1 mg/dL in the 66–90 mg/dL range (*n* = 43), and 1.5 ± 6.0 mg/dL in the 56–65 mg/dL range (*n* = 77; Figure 7). Interstitial glucose concentration instead overestimated BG by a mean of 6.3 ± 5.7 mg/dL in the 46–55 mg/dL range (*n* = 95) and 15.7 ± 5.6 mg/dL in the < 45 mg/dL range (*n* = 27; Figure 7). Overt signs of neuroglycopenia were not observed. Adverse events were characterized by five episodes of vomiting (from collective data at all rates of glycemic change). One episode occurred during severe hypoglycemia (40 mg/dL), 2 during marked hypoglycemia (52–57 mg/dL), and 2 during moderate hypoglycemia (60 mg/dL).

## 4 Discussion

Our results showed good clinical accuracy of FSL3 during hypo‐ and euglycemia. The use of CGMS represents a paradigm shift in the management of veterinary diabetic patients [2]. Several studies have described the accuracy of previous generations of FSL in cats [4, 10, 11, 12, 14], but none have evaluated the performance of FSL3. The accuracy of FSL3 in cats during hypoglycemia and euglycemia is reported here with 474 paired BG‐IG measurements, 223 of which were in the hypoglycemic range (BG < 60 mg/dL), including 160 paired results recorded during periods of stable glycemia. A strong positive correlation between IG and BG was observed during periods of stable glycemia. However, when considering the entire dataset, including both stable and unstable BG, we found a moderate correlation. Our results are in agreement with a previous study evaluating the FSL2 in hypo‐ and euglycemic cats [14]. Direct comparison with other studies is limited by differences in hypoglycemic sample size, reference method of glucose measurement (vPBGM vs. hexokinase‐based laboratory assays), study populations (client‐owned diabetic vs. purpose‐bred cats), and differences in sampling environments and frequencies (home‐based, hospital‐based, or controlled research laboratory settings) [4, 10, 11, 12].

Similar to previous veterinary CGM studies utilizing ISO 15197:2013 guidelines, standards for clinical (but not analytical) accuracy were met in our study. Only 79% of the data points met the analytical accuracy criteria outlined by the ISO 15197:2013 standards. Our results are comparable to previous studies in cats, with reported analytical accuracy that ranged from 42% to 73% [10, 11, 12, 14]. Parkes EGA demonstrated strong clinical accuracy, with 100% and 99% of results classified within zones A and B during periods of stable and unstable glycemia, respectively. The ISO 15197:2013 guidelines require that PBGM measurements be compared to a standardized reference method. However, these standards are tailored for comparisons within a single compartment, typically blood, and might not be fully applicable for cross‐compartmental comparisons (i.e., between blood and interstitial fluid) because of the physiological differences between these compartments.

Analytical accuracy is not expected for CGMS and generally is not met because IG and BG measure glucose in different compartments [10, 11, 12, 14]. Given the absence of well‐established criteria for assessing glucose measurement accuracy in interstitial fluid, the ISO standards for PBGMs offer a useful proxy, helping to identify CGMS devices that closely adhere to accuracy criteria and that are not dangerous for the individual's health. With these limitations in mind, the FSL3 can be considered suitable for clinical use in cats, demonstrating performance similar to the FSL2 in the euglycemic and hypoglycemic ranges. Despite the good clinical accuracy of earlier FSL models, premature sensor detachment remains one of the most commonly reported complications in diabetic cats, with median sensor activity ranging from 5 to 10 days [4, 10, 11, 12]. Additionally, in a recent survey, one of the most important concerns expressed by owners of diabetic pets, particularly those of diabetic cats, was the limited FSL lifespan [19]. The smaller size of the FSL3 might improve both tolerability and adherence to long‐term CGMS use in cats. In our study, it was not possible to assess the duration and tolerability of the sensor in the experimental setting, and additional studies are needed in diabetic cats.

Our data suggest a small and proportional bias in IG results in the hypoglycemic range. As previously reported in cats for the FSL2 [14], IG measured by the FSL3 tends to underestimate BG in the euglycemic range in cats, with the difference decreasing as BG decreases. However, in cases of marked hypoglycemia, IG tends to overestimate BG in healthy cats. Based on our findings, IG results in the severely hypoglycemic range (< 45 mg/dL) should be approached with caution and interpreted as potentially representing BG at an equal or lower concentration. Hypoglycemia is a major limiting factor in the management of DM in patients receiving insulin treatment. In surveys that investigated the quality of life of owners of diabetic pets, as well as the perceived quality of life of their diabetic pets, owners' fears of hypoglycemia had one of the largest negative impacts on their quality of life [8, 9]. The CGMS improves the detection of hypoglycemic episodes [3] and might decrease the frequency of clinical hypoglycemia when used for insulin dose titration [20]. In our study, the experimentally induced hypoglycemia offered a valuable opportunity to ethically and efficiently collect a large amount of IG data within this glycemic range. In a clinical context, discrepancies between BG and IG can be partially attributed to the time necessary for glucose concentrations to equilibrate between the blood and interstitial fluid. By maintaining a stable BG using the glucose clamp technique, we were able to more precisely evaluate the bias in IG measurement relative to BG, minimizing the confounding effects of the lag time required for equilibrium between the two compartments. This lag in detecting changes in glucose concentrations likely results from both biological and technical considerations associated with the measurement of IG. First, there is a temporal gap between fluctuations in BG and the time required for these changes to be reflected in the interstitial fluid. This gap is influenced by factors such as glucose transport across endothelial barriers and the concentration gradient [11]. Second, the diffusion distance from blood vessels to the sensor adds to lag time, as well as algorithmic delay in reporting the IG. Previously reported lag times vary widely based on the method used to induce a change in BG, whether BG is increasing or decreasing, the CGMS used, and the definition of lag time [11, 21]. In cats, the rapid administration of high‐dose IV glucose (0.5 g/kg) leads to a delay of 5–15 min before an initial increase in IG is observed, and 30–45 min until peak IG [11]. This delay is further extended in patients with decreased interstitial tissue perfusion, such as elderly or dehydrated animals [11, 22], as well as when BG fluctuations are rapid and of significant magnitude [23]. The cats in our study were normally hydrated and within the young‐adult age range. Hence, BG‐IG lag time obtained in dehydrated and older cats may require additional evaluations. However, age and hydration status are not expected to affect the accuracy of the system under stable conditions.

In diabetic cats, BG might vary substantially both throughout the day and across consecutive days [24]. Several factors contribute to BG variability, including inconsistent insulin absorption and degradation, variable numbers of residual β cells, technical issues with insulin administration, concurrent illnesses, and variable levels of stress, among others [24]. In contrast, in healthy cats, BG exhibits minimal variation from day‐to‐day. In our study, the magnitude of BG changes was confined to a narrow hypoglycemic range, and sympathetic responses that could lead to unpredictable and rapid BG fluctuations were minimized using restraint‐free handling and acclimatization to study personnel and laboratory conditions. Therefore, in clinical practice, the FSL3 is not optimal for estimating BG at the moment of its measurement. However, it is exactly because of substantial glucose variability in diabetics that clinically measuring IG in a continuous manner is superior to measuring BG in predicting future BG and making treatment decisions. Therefore, with the exception of confirming specific measurements (e.g., when a diagnosis of neuroglycopenia needs to be confirmed) there is no advantage to confirming IG readings with BG, even when the rate of IG change is high. In addition, with the advent of sodium‐glucose cotransporter 2 (SGLT2) inhibitors in the management of DM in cats, a lower glucose variability is anticipated [25, 26]. Therefore, the performance of the FSL3, as evaluated in our study under experimental conditions, is likely to be appropriate for these patients.

Vomiting was the only adverse effect observed in response to hypoglycemia in our population of fasted, healthy cats, consistent with findings from our previous study [14]. In that study, vomiting did not appear to correlate with the severity of hypoglycemia, because it was observed during both moderate (60 mg/dL) and severe (40 mg/dL) hypoglycemia. Vomiting could be considered a warning sign prompting a caregiver to check the cat's BG. However, because of the experimental nature of our study, definitive conclusions cannot be drawn, and the relationship between vomiting and hypoglycemia in cats requires further investigation.

Limitations of our study include the use of non‐diabetic cats with experimentally induced hypoglycemia for data collection. Hypoglycemia was achieved using infusion of regular insulin and leading to rapid fluctuations in BG, which may not fully replicate the glycemic patterns seen with the intermediate‐ or long‐acting insulin formulations typically used in a clinical setting. Although the physiological mechanisms underlying the discrepancy between BG and IG are likely consistent regardless of the cause of hypoglycemia, further research is needed to validate our findings in diabetic cats and to assess the accuracy of the device within the hyperglycemic range. Moreover, the experimental design of our study did not allow for the evaluation of certain logistical challenges related to the use of the FSL3 in clinical practice. Notably, the FSL3 sensor can only be paired with a single device, which may pose practical difficulties in hospitalized patients. This limitation could be mitigated by utilizing the FreeStyle Libre 3 dedicated reader, which can be employed by both healthcare providers and pet owners. However, the availability of this reader remains limited, and it is not yet accessible in all regions worldwide.

In conclusion, the FSL3 provides clinically accurate measurements in the euglycemic and hypoglycemic ranges in cats. Clinical interventions prompted by IG measurements within the hypoglycemic range can have clinically relevant consequences. Therefore, recognizing the proportional glycemic‐dependent bias associated with FSL3 IG allows clinicians to enhance the safety of its application. Clinicians should be cautioned that although the FSL3 tends to underestimate BG in most of the euglycemic range in cats, it may overestimate BG in hypoglycemic ranges < 55 mg/dL. Interstitial glucose concentration readings in the severely hypoglycemic range should be approached with caution, and it might be advisable to assess BG using a validated vPBGM to confirm the FSL3 results. The smaller size of the FSL3 could enhance tolerability and extend sensor lifespan, facilitating the monitoring of diabetic cats. Additional studies are needed to assess the performance and tolerability of the FSL3 in diabetic cats.

## Disclosure

Authors declare no off‐label use of antimicrobials.

## Ethics Statement

Approved by the University of Florida Institutional Animal Care and Use Committee (protocol number 202300000345). Authors declare human ethics approval was not needed.

## Conflicts of Interest

The authors declare no conflicts of interest.
