# Erratum for “Accuracy of the Freestyle Libre 3 Continuous Glucose Monitoring System in Hypo‐ and Euglycemic Cats”

**DOI:** 10.1111/jvim.70085

**Published:** 2025-04-11

A. M. Tardo , C. Crews , J. Mott , L. T. Porter , C. Adin , and C. Gilor , “Accuracy of the Freestyle Libre 3 Continuous Glucose Monitoring System in Hypo‐ and Euglycemic Cats,” Journal of Veterinary Internal Medicine 39 (2025): e70048.40105400 10.1111/jvim.70048PMC11921137

In the above mentioned article, Reference 14 is incorrect. The correct Reference 14 should be as follows.

“A. S. Berg, C. D. Crews, C. Adin, et al., “Assessment of the FreeStyle Libre 2 interstitial glucose monitor in hypo‐ and euglycemic cats,” *J Vet Intern Med*. 2023: 37(5):1703–1709. https://doi:10.1111/jvim.16820.”

We apologize for this error.

